# “Why am I still suffering?”: Experience of long-term fatigue and neurocognitive changes in oropharyngeal cancer survivors following (chemo)radiotherapy

**DOI:** 10.1016/j.tipsro.2024.100241

**Published:** 2024-03-08

**Authors:** Zsuzsanna Iyizoba-Ebozue, Emma Nicklin, James Price, Robin Prestwich, Sarah Brown, Emma Hall, John Lilley, Matthew Lowe, David J Thomson, Finbar Slevin, Louise Murray, Florien Boele

**Affiliations:** a Department of Clinical Oncology, Leeds Cancer Centre, Leeds, UK; b Leeds Institute of Medical Research, University of Leeds, Leeds, UK; c Department of Clinical Oncology, The Christie NHS Foundation Trust, Manchester, UK; d Leeds Cancer Research UK Clinical Trials Unit, Leeds Institute of Clinical Trials Research; e The Institute of Cancer Research, London, UK; f Department of Radiotherapy Physics, Leeds Cancer Centre, Leeds, UK; g Christie Medical Physics and Engineering, The Christie NHS Foundation Trust, Manchester, UK; h Manchester Academic Health Sciences Centre, Faculty of Biology, Medicine and Health, The University of Manchester, Manchester, UK

**Keywords:** Neurocognition, Fatigue, Oropharyngeal Cancer, Quality of Life, Late effects, Radiotherapy

## Abstract

**Background:**

Late effects of cancer treatment, such as neurocognitive deficits and fatigue, can be debilitating. Other than head and neck–specific functional deficits such as impairments in swallowing and speech, little is known about survivorship after oropharyngeal cancer. This study examines the lived experience of fatigue and neurocognitive deficits in survivors of oropharyngeal squamous cell cancer and impact on their daily lives.

**Methods:**

This work is part of the multicentre mixed method ROC-oN study (Radiotherapy for Oropharyngeal Cancer and impact on Neurocognition), evaluating fatigue and neurocognitive function in patients following radiotherapy +/- chemotherapy for oropharyngeal cancer and impact on quality of life. Semi-structured interviews were conducted in adults treated with radiotherapy (+/-chemotherapy) for oropharyngeal squamous cell carcinoma >/=24 months from completing treatment. Reflexive thematic analysis performed.

**Results:**

21 interviews (11 men and 10 women; median age 58 years and median time post-treatment 5 years) were conducted and analysed, yielding six themes: (1) *unexpected burden of fatigue, (2) noticing changes in neurocognitive function, (3) the new normal, (4) navigating changes, (5)insufficient awareness and (6)required support.* Participants described fatigue that persisted beyond the acute post-treatment period and changes in neurocognitive abilities across several domains. Paid and unpaid work, emotions and mood were impacted. Participants described navigating the new normal by adopting self-management strategies and accepting external support. They reported lack of recognition of these late effects, being poorly informed and being unprepared. Follow-up services were thought to be inadequate.

**Conclusions:**

Fatigue and neurocognitive impairment were frequently experienced by survivors of oropharyngeal cancer, at least two years after treatment. Patients felt ill-prepared for these late sequelae, highlighting opportunities for improvement of patient information and support services.

## Highlights

• Limited data exist on survivorship after oropharyngeal cancer with existing literature focused predominantly on head and neck–specific physical functional deficits.
• This study examines the lived experience of fatigue and neurocognitive deficits in survivors of oropharyngeal squamous cell cancer and impact on their daily lives.
• Cognitive fatigue and neurocognitive impairment across several domains were frequently experienced by survivors of oropharyngeal cancer (at least two years post-treatment) affecting quality of life.
• Survivors felt ill-prepared for these late sequelae, highlighting opportunities for the improvement of patient information and support services.

## Introduction

Head and neck cancers (HNC) represent a heterogenous group of diseases and account for more than 660,000 new cases annually [1], [2]. Carcinogenic human papilloma virus (HPV) has resulted in an increased incidence of oropharyngeal cancer (OPC) with most cases observed in patients < 65 years of age and associated with a more favourable prognosis [3], [4], [5], [6], [7]. Treatment is generally multimodal, with concurrent chemoradiotherapy (CRT) being standard of care [8], [2]. Late effects of CRT are a key survivorship issue. These include permanent loss of saliva, swallow dysfunction, dysgeusia, lymphoedema, skin fibrosis, and neuropathy amongst other toxicities [9], [10], [11]. Consequently, patients treated for OPC, can face years of survival with significantly impaired quality of life(QoL)due to late effects of curative treatment [12].

The National Comprehensive Cancer Network (NCCN) defines fatigue as “*a distressing, persistent, subjective sense of physical, emotional, and/or cognitive tiredness or exhaustion related to cancer or cancer treatment that is not proportional to recent activity and interferes with usual functioning”* [13]. It is one of the most prevalent and distressing late effects of cancer treatment [14], [15]. Chronic fatigue (CF) is fatigue lasting for six months or more [16], [17]. In a cross-sectional study of patients with HNC, a moderate-to-severe fatigue rate of 18 % at least 1 year after RT was reported [18]. This is high in comparison to a prevalence rate in the general population of approximately 0.5–1 % [19], [20]. Fatigue appears to remain an issue throughout survivorship, with a prospective longitudinal study in HNC survivors showing ongoing fatigue from 1 to 5 years of follow-up, without demonstration of recovery [21].

During head and neck radiotherapy (RT) normal brain tissues may receive a low radiation dose, and this could have a detrimental effect on neurocognitive function [22], [23], [24]. Several small retrospective HNC series report neurocognitive dysfunction within 2 years of treatment [25], [26], [27]. Observed cognitive decline appears to persist > 5 years post-treatment [28], [29], [30] and memory problems, fatigue, anxiety and reduced QoL are reported in HNC survivors [29], [30], [31], [32], [33], [34], [35], [36]. These studies are limited by small sample sizes, heterogeneity of HNC patients sampled or a sole focus on nasopharyngeal cancer. Furthermore, neurocognition was often assessed using screening tools which lack sensitivity and domain-specific information. In addition, previous studies have mostly had quantitative methodology [36], which although valuable, lacks contextual information on how late effects may impact on patients’ everyday lives.

To obtain greater insight into patient quality of life, ROC-oN (Radiotherapy for Oropharyngeal Cancer and impact on Neurocognition reference 22/WM/0207), a mixed method cross sectional study evaluating fatigue and neurocognitive function in patients following RT +/- chemotherapy for OPC and impact on QoL was carried out.Here, we report on the qualitative arm of ROC-oN performed to gain a better understanding of survivors’ perspectives and experiences.

## Methods

### Study design

This qualitative investigation is part of a larger multicentre study, approved by the West Midlands Research Ethics Committee in October 2022 (22/WM/0207). The full study involved patients with OPC being invited to complete a quantitative survey and an online unsupervised cognitive test battery, after which a subset of participants were invited for qualitative interviews. Here, we report on the qualitative findings only. We used semi-structured interviews and thematic analysis, supported by a reflexive approach [37], [38], [39].

### Setting and participants

Patients treated for OPC with primary RT (+/-chemotherapy) in Leeds Cancer Centre or The Christie NHS Foundation Trust from 2010 to 2020, with ≥ 24 months follow-up and disease free at time of recruitment, were identified from existing institutional databases and sent postal invitations. Exclusion criteria were inability to consent, limited English language proficiency, surgery prior or after RT.

Following completion of quantitative data collection, participants indicated their willingness to be contacted for interview. To strike a balance between meaningful representation and resource constraints, approximately 10 % of the total eligible study participants were approached. Convenience and purposive sampling techniques were adopted to obtain a varied participant sample by age (at treatment and time of study), tumour subsite (e.g., tonsil, base of tongue), Tumour-Node-Metastasis (TNM) staging, HPV status, time interval since end of treatment, level of education and employment status.

Interviews were conducted March to June 2023. Verbal and written study information was provided and written or recorded verbal consent was obtained.

### Data collection

Interviews were conducted by telephone or online video platform, dependent on participant preference. Interviews were semi-structured, exploring fatigue and neurocognition since treatment and other late effects.

Neurocognitive deficits referred to impaired ability to pay attention, concentrate, remember, follow conversations, multitask and/or a general sense of limitations in linguistic and arithmetic capabilities [40]. The NCCN definition of fatigue was adopted [13]. As both are multidimensional constructs, there is some overlap between the emotional and cognitive aspects of fatigue and neurocognitive function, and fatigue will impact on neurocognitive performance. For the purpose of this study, emotional/cognitive fatigue was considered a psychobiological state caused by demanding neurocognitive tasks, presenting as a feeling of tiredness, lack of energy, decreased motivation and alertness [41], [42], while physical fatigue referred to the physical sensation of tiredness and impact of fatigue on physical activity [13], [43].

Interviews were conducted with participants one-to-one by one of two researchers with different backgrounds (ZIE a clinician and EN a sociologist and experienced qualitative researcher). An interview guide (supplementary information 1) was used. Reflective notes were made after interviews and regular discussions held within the research team.

### Analysis

Data collection was completed when theoretical saturation was achieved and sufficient depth of understanding attained in order to address the research questions [44]. Interviews were transcribed verbatim and analysed using reflexive thematic analysis [37], [38] taking into account our role as researchers in the study. The study employed a paradigmatic framework of interpretivism and constructivism. We aimed to explore patients subjective experience of fatigue and neurocognitive abilities after OPC treatment, whilst acknowledging the importance of researcher influence in such interpretations. No specialised qualitative software was used.

All transcripts were read and then coded (ZIE), with coding being inductive to fully understand participant experiences but also deductive to find data to address the research objectives. Initial codes were subsequently categorised into potential sub-themes and themes (coding summary table in supplementary information 2) and coding regularly discussed with research team (EN, FB, LM).

A second coder (EN) was used to sense-check ideas and explore multiple assumptions or interpretations of the data [37], [38], [39], thereby developing richer interpretations of meaning, instead of striving to achieve consensus of meaning.

## Results

A total of 257 ROC-oN study participants expressed an interest in being interviewed, of whom 33 were approached and 21 were interviewed (Table 1 shows participant characteristics). Interviews took on average 52 min (range 41–70). 11 men and 10 women participated, median age was 58 years and median time post-treatment was 5 years (inter quartile range 4–8). All participants received CRT. Interviews produced six themes with sub-themes, as discussed below.

**Table 1:** Participant characteristics.

Participant number	Sex	Age	Time since EOT	Sub-Site	TNM (8)	HPV	Working	Education
1	M	66	5yrs	Tonsil	T1N2a	–	No-retired	A level
2	F	50	5yrs	BOT	T4N1	+	–	Diploma
3	F	61	3yrs	Tonsil	T1N2	+	No-retired	NVQ
4	F	58	3yrs	Tonsil	T1N1	+	Yes-full time	NVQ
5	F	66	5yrs	Tonsil	T2N1	+	No-retired	School
6	M	50	3yrs	Tonsil	T1N1	–	Yes-full time	Diploma
7	F	59	9yrs	BOT	T2N2	+	No-statutory sick pay	School
8	M	71	5yrs	Tonsil	T3N0	+	Yes-self employed	School
9	M	58	4yrs	Tonsil	T3N1	+	Yes-full time	6th Form
10	F	52	9yrs	Tonsil	T2N2	+	Yes -full time	Degree/Higher education
11	M	58	5yrs	Vallecula	T2N2	+	No-cannot work	Degree/Higher education
12	M	62	3yrs	Tonsil	T1N2	+	Yes-part time	A level
13	M	72	6yrs	Tonsil	T4N2	+	No-retired	GCSE
14	F	65	5yrs	Tonsil	T3N0	–	Yes-part time	A level
15	M	51	8yrs	BOT	T3N0	+	Yes-full time	NVQ
16	F	72	11yrs	Tonsil	T2N1	–	No-retired	No formal
17	F	61	4yrs	Tonsil	T1N1	+	No-retired	GCSE
18	F	56	9yrs	Tonsil	T4N0	–	No-retired due to ill health	Foundation degree
19	M	47	4yrs	Tonsil	T2N1	_+_	Yes-part time	–
20	M	64	5yrs	BOT	T2N1	+	No-due to ill health	A level
21	M	61	8yrs	Tonsil	T3N2	+	Yes-full time	No formal

Base of Tongue (BOT), Tumour Node Metastasis (TNM), Human papilloma virus (HPV), End of treatment (EOT), National Vocational Qualification (NVQ), General Certificate of Secondary Education (GCSE), Working > 30 h/week (full time), Working < 30 h/week (part time), Advanced Level Qualification (A-level

**Unexpected burden of fatigue:** “*I did not expect to still feel quite so tired now at 2*–*3 years down the road”*

Participants all experienced fatigue during and/or in the weeks after RT. For most this was incapacitating and like no previous experience. Fatigue gradually improved 4–6 months post-treatment but for some, fatigue persisted for several years*.* In most participants with CF, it was mild to moderate, however, in a few cases fatigue was severe. Irrespective of the degree of CF, participants did not confuse this with normal tiredness and reported they never returned to baseline (Table 2, section A, Comment 2).

**Table 2:** Themes, sub-themes, and illustrative quotes.

*Themes*	*Sub-theme*	*Illustrative Quotes*
A. UNEXPECTED BURDEN OF PERSISTENT FATIGUE	***Fatigue persisting beyond acute period***	1. “I just feel very tired ……I've tried not to go on about it, but it is a problem, yes” [Participant 14, female, 65 yr]. 2. “tiredness and fatigue are two different things…. everybody gets tired from time-to-time, and you either take a day off or you have a bit more sleep, but fatigue it’s just a totally different animal” [Participant 12, male, 62yrs].
	***Types of fatigue experienced***	3. “I felt drained… I have never experienced such a lack of energy, to the extent where I rang one of my sons up…and said, oh, will you turn the music on please, and the Christmas lights on, which he could do remotely, I just couldn't get off the sofa to do that” [Participant 9, male 58yrs] 4. “a lot of it is memory-related and kind of brain power” [Participant 9, male, 58yrs] 5. “I have the foggy days, foggy days, the foggy times when I’m tired” [Participant 4, female, 58yrs].
	***Causative uncertainty***	6. “the thought I have going through my mind is, is part of this just normal getting a little bit older or is this partly because of the treatment and I honestly don’t have the answer to that” [Participant 6, male, 50yrs] 7. “I recognise that I do get a bit tired, and I guess it’s difficult because obviously since treatment I’ve gone through the menopause, ……. the more time goes on I probably relate it less to treatment and more to life in general, menopause and those sort of things” [Participant 2, female, 50yrs] 8. “managed to eventually get diagnosed with an underactive thyroid, [got] on Thyroxine, and gradually….…...started feeling better” [Participant 19, male, 47yrs].
B. NOTICING CHANGES IN NEUROCOGNITIVE PERFORMANCE	***Timing of neurocognitive changes***	1. “my nervous system somehow, I don’t feel as robust as it was, but …. I don’t have the evidence for that” [Participant 15, male, 51yrs 2. “I wouldn’t say it was at the beginning…. I would say from like that second year onwards probably when I was slightly back to normal, I probably recognised it then more” [Participant 2, female, 50yrs]. 3. 3. “It was pretty quick to be honest, it was within I would say a month or two that I realised that I couldn’t work as quick as I do, so it was very, very quick” [Participant 12, male, 62yrs ].
	***Affected Domains(memory, attention, and processing speed)***	4. “my memory got bad, even simple tasks……. I couldn’t remember to make an appointment, even though I made it I didn’t remember that I’d made it… I’ll read something and forget what I’ve read” [Participant 10, female, 52yrs]. 5. “even though I’d known them for 12 years I didn’t know their names anymore” [Participant 21, male, 61yrs] 6. “my memory has been very much affected, particularly the short-term memory more so than the long-term memory …. people would give you information, and which you needed to recall in, you know, a few minutes after that, you’d think “what they said again?”” [Participant 18, female, 56yrs] 7. “I find it difficult enough to read a text message and to stay focused on it long enough to make the relative reply” [Participant 11, male, 58yrs]. 8. “taking onboard the information, processing that and getting to the decision as quick as I need to is not where I should be really” [Participant 12, male, 62yrs]. 9. “I have found just recently, and this is probably within the last 12 months, I’ve sort of, I’m not aware of danger or space, I know this will sound ridiculous, but I’ll do something and ……. then I’ll turn round, and I’ll just knock it flying and I’ll think, well I’ve just done that, how couldn’t I remember I feel, that I sort of, I’m not aware of danger or space” [Participant 16, female, 72yrs]
C. *THE NEW NORMAL*	***Employment and paid work***	1. “I made the choice not last year, the year before, …. I’d go part-time” [Participant 19, male, 47yrs] 2. “now I think my best bet is to have a job where I can work from home, where I can rest when I need to, but I still do my job” [Participant 10, female, 52yrs] 3. “I ended up being ill health retired from my role at work, and I haven't worked since…… it’s eight years now.” [Participant 18, female, 56yrs]. 4. “I’ve familiarised with people and the graphics again, I’m doing the same job all the time which I requested to do, …. so, I can sort of like become familiar with me routes, the people I’m dealing with and so on and so forth” [Participant 21, male, 61yrs]. 5. “I had to change the deployment room that I was working in, … rather than make a mistake” [Participant 12, male, 62yrs] 6. “I'm having to double check and double check work, which is time consuming and frustrating”. [Participant 9, male, 58yrs]
	***Leisure and social function***	7. “it’s really taken the joy out of what I wanted to do, and which was something of a pleasure, as it’s really challenging”. [Participant 19, male, 47yrs] 8. “I just don't want to do anything. I just……lack confidence. I don't drive anymore” [Participant 18, female, 56yrs]. 9. “meeting friends, I might not have the energy, …… so I kind of tend to withdraw at these times and it's like I become really unsociable, even though I'm not”. [Participant 14, female,65yrs]
	***Mood and emotions***	10. “I’m more anxious than I used to be about things and making sure things are in place and things are right” [Participant 4, female, 58yrs]. 11. “the frustrating part was I was very sharp; I was a manager. I was in charge, I used to run things, I was always in control, and I’ve gone from [one]extreme to the other.” [Participant 21, male, 61yrs ] 12. “you just learn to manage around it, don’t you, …. you can’t be mended so you just have to get on, don’t you”? [Participant 4, female, 58yrs] 13. 13. “I’m not massively worried about it but I am a bit mindful of it in case it gets any worse, …. I remember thinking, I don’t think I was this bad at this before” [Participant 6, male, 50yrs].
D. NAVIGATING CHANGES	***Self-management and coping***	1. “Try and split your day up a little bit, instead of trying to do everything in one go…… Sleep when you need to and don’t be upset about it and don’t be worried about what other people think.” [Participant 3, female, 61yrs]. 2. “I just slowly work through it, ….and if I have found things difficult, I’ve asked for the extra time to do it” [Participant 14, female, 65yrs]. 3. “Dividing work a bit more and not taking as big, such a big slice of work…… “I’m like just do one thing at a time, ………. just stop and do one thing” [Participant 6, male, 50yrs] 4. “I make a lot of lists of things. I use my phone for lists, I use pen and paper for lists, ….to make sure I’m keeping myself on track because I can forget things very, very easily” [Participant 6, male, 50yrs]. 5. 5. “it’s very important that I eat at the right times, you know at the right intervals, ……. otherwise, my mind sort of, well I think my head’s not as focused”. [Participant 15, male, 51yrs].
	***Social support (family and work colleagues)***	6. “I get a lot of support at work, I’ve got like a PA now that helps me to do all the admin and stuff, …………they accommodate me by saying “oh well we know that you need support”” [Participant 10, female, 52yrs]. 7. “I’ve got a very understanding team and they’re brilliant, but you can’t… in that sort of world you can’t expect people to make allowances for too long” [Participant 12, male, 62yrs] 8. “I utilise my wife as much as I can [laughs]” [Participant 9, male, 58yrs].
E. INSUFFICIENT AWARENESS	***Late effects***	1. “When I think about what the effects afterwards, that probably had more impact on me than the actual treatment” [Participant 2, female, 50yrs]. 2. “I think the positive attitude is [key], but people are different and people …. I don’t know how you make somebody positive when it is a pretty dire situation…… [Participant 1, male, 66yrs]
	***Unprepared***	3. “I had no idea the impact long-term. you’ve got no idea what’s coming really, …… I could have never in my wildest dream[imagine], the impact it has on your life” [Participant 16, female, 72yrs]. 4. “I was aware, but I didn’t realise that it[fatigue] will last this long if you know what I’m saying? I just thought it would be like maybe for the first couple of years and then you’d probably get back to normal, but I’ve never been the same since the treatment” [Participant 10, female, 52yrs]. 5. “There was lots about the physical symptoms, the fatigue, the beard falling out, the skin, …. but I would say I don’t think it was mentioned that cognition would be potentially changed, or memory would be impacted” [Participant 6, male, 50yrs] 6. “that [neurocognitive deficit] had never been mentioned, in fact the side-effects of the radiotherapy really didn’t get discussed in great detail apart from, you know, it’ll hurt your throat a bit” [Participant 8, male, 71yrs].
	***Importance of good information provision***	7. “You know, we’re still three, four, five years on and you’re still suffering. I only got signed off September last year. But, at the end of the day, throughout all those five years, you have ongoing issues through diet, some people have fatigue, etc. They do play it down slightly” [Participant 13,male, 72yrs]. 8. “I mean obviously you don’t want to think things will change more but if it was within the list of my things that were given to me as this may, you know, like you will, you will have a dry mouth, you will struggle more harder with swallowing some foods, you may have cognitive problems in the future. Then at least you can prepare yourself a bit for it, I think” [Participant 4, female, 58yrs] 9. “To recognise that it [neurocognitive changes] is normal, again you’re not losing your marbles. And I think just to try and to focus on ways that you can get round it and help……. to be aware of that and to perhaps, to think about strategies at that time would probably be useful” [Participant 2, female, 50yrs] 10. 10. “since that letter come through the post [invitation to participate in the ROC-oN study] ……[saying] the people who have maybe had this treatment maybe could have had this [neurocognitive impairment]. Since they’ve all read the letter and it’s there in black and white, [they have] been completely understanding. It’s been a blessing, really” [Participant 21, male, 61yrs].
REQUIRED SUPPORT	***Seeking support***	1. “you don’t want to burden your family and talk, after a couple of years you try and pretend everything’s fine …. after all this time it’s part of my life, it’s my normal but I don’t talk about it because, you know, your family don’t want to listen to it, they’re fed up with hearing or, you know, you just pretend everything’s fine, so it’s a tough one really” [Participant 16, female, 72yrs] 2. “It’s difficult to talk to GPs about something like this because it’s not their specialism. You need someone who kind of…Who gets it” [Participant 8,male, 71yrs]. 3. “No, I didn't really think that it would be recognised, you know, erm… because I think it should be, but I don't even think doctors are fully aware of, you know, the aftereffects, and I think that's where the problem stems from, because it actually puts you off going to see the doctor” [Participant 14, female, 65yrs ].
	***Unsatisfactory Long-term follow-up***	4. “I think there's not a great deal of support afterwards for head and neck cancer patients” [Participant 18, female, 56yrs]. 5. “And it was actually Macmillan who got my last lot of appointments because I hadn’t seen the specialist for a year, I hadn’t been able, when I speak to him about anything he just says “oh it’s normal, you’ll have it for the rest of your life, nothing you can do about it” …… ..he’s not interested, he doesn’t care how I feel, he just wants to know if it’s come back and if they can give me more treatment”[Participant 11, male, 58yrs].

Whilst acute fatigue appeared present more in the physical domain, CF appeared most in the dimension of emotional/cognitive fatigue, often described as “brain fog” or being “fuzzy headed” (Table 2, A4 &5). One participant described being more physically active and going to the gym but being burdened by a lack of “emotional connection”, leaving them unable to commit to work or pursue career progression. Other participants expressed difficulty in finding motivation to take on complex tasks at work, leading on workplace projects and struggling with working on a computer.

Participants with CF post-treatment were unsure what to attribute symptoms to. They felt fatigue could be due to aging, fluctuating hormone levels, or hypothyroidism. Participants thought getting older might have caused some decline in endurance and energy levels and female participants also mentioned normal physiological endocrine changes such as menopause could impact fatigue. Yet, participants were not convinced these factors fully explained their CF and questioned if it could be due to their OPC treatment (Table 2, A6).

**Noticing changes in neurocognitive performance*:*** *“Is it just me? Why am I doing this, what’s going on here?”*

Some participants reported noticing unexplained changes in neurocognitive functioning after the first-year post-treatment which became more apparent in the longer term (Table 2, section B, comment 2). One participant reported experiencing neurocognitive deterioration within the first few weeks of treatment completion.

Amongst participants, change in neurocognitive performance was noticed across several domains. Memory was the most commonly and most severely affected domain. This was mild for most participants: they expressed they struggled to remember names of people, items in a food order or occasional appointments. However, for some, severe memory issues were described, e.g., being unable to remember people they had known for over a decade (Table 2, B5). Most participants expressed issues with ‘short-term’ memory; struggling to recall facts shortly after information had been received.

Attention, especially sustained attention, was another commonly affected domain, with participants struggling to concentrate on simple tasks such as responding to telephone messages, watching a film, or reading a book. Changes in processing speed were reported as well: taking onboard information, processing it, and reaching a decision was much slower than pre-treatment (Table 2, B8). Participants also reported impairment in planning skills when taking part in handiwork.

In one participant who reported no visual problems, impaired spatial awareness was suggested, with a lack of awareness for “danger or space” expressed. This led to an accident in which the participant sustained significant physical injury. It is possible that this could be linked to impaired memory of spatial information.

**The new normal:** “*work has certainly changed, and I get kind of concerned”*

Participants who returned to employment after treatment reported fatigue was exacerbated by the demands of work and neurocognitive changes made work challenging. For example, participants described not being able to go over work emails quickly, having to double check their work several times and struggling to deal with high level and load of information (Table 2, C4&6). This consequently often translated to a change in work pattern (going part-time) and, in some cases a change in job role (i.e., stepping down from managerial roles). For a few participants despite modifications to work pattern and job role, they described being to cope with the physical and psychological demands of work and were unable to retain employment (Table 2, C3). Some participants stated, re-learning relevant activities and skills fostered a feeling of competency despite impaired neurocognitive performance.

Participants said neurocognitive changes negatively influenced engagement with hobbies and interests (e.g., reading, handicrafts). Instrumental activities of daily life such as driving, which were effortless pre- treatment, became difficult for some. Relationships with family and friends were reported to suffer strain in some cases, leading to social isolation (Table 2, C9).

Participants also linked impairment in neurocognitive function and fatigue to low mood and anxiety. Sometimes this exacerbated anxiety around cancer recurrence. Inability to perform at a desired level led to frustration, however some participants maintained a positive attitude and persevered. Participants wanted to be aware and keep track of further neurocognitive changes.

**Navigating changes:** “*I have learnt to manage what I have to do in my own way”*

To manage memory issues, keeping notes, making lists and routines were the most favoured strategies reported by participants. These memory aids appeared sufficient for participants with mild memory loss. To self-manage cognitive fatigue and neurocognitive deficits, participants also expressed they had to take rests or move through the day at a slower pace. In addition, for some, dividing tasks to optimise participation in desired daily life activities and a different approach to organising tasks was required with more mentally taxing tasks done in the morning (tabl2 2, section D, comment 1–3). Some participants also highlighted adopting healthy eating habits as a strategy to manage fatigue.

Most participants with CF or neurocognitive changes described receiving support from work colleagues and that this was vital to remaining in employment. Many expressed increased reliance on family (Table 2, D8). Participants were grateful for their family’s understanding and support, but also aware of some loss of independence and feeling like an additional burden on their family.

Some participants expressed a positive outlook and this positive psychology had significant impact on how they managed late effects and promoted proactive measures to protect health.

**Insufficient awareness:** *“Six years down the line, this is why I am thinking why am I still suffering? I wish they had told me it would be as long-term as it is”*

Participants experienced a wide range of physical late effects from treatment (supplementary Table 2), to varying degrees. These late effects presented difficult challenges to participants, e.g., impaired salivary gland function, problems with swallowing and oral pain served as a daily reminder of treatment, impacting physical nutritional needs, making eating an obligation and impacting negatively on social aspects of eating.

Many participants described being generally unprepared for the late consequences of treatment (Table 2, section E, comment 3). Fatigue was mentioned but not emphasised during the pre-treatment consent process, making it easy to underestimate its long-term impact. One participant recalled chemotherapy-related brain fog was mentioned but others had no recollection of long-term changes in neurocognition being mentioned as a potential late effect(Table 2 , E5). This meant many had to source information for themselves on the internet.

Participants said they would like to have been better informed and equipped to recognise the late effects they are at risk of developing (Table 2, E8&9). They expressed being more informed of CF and possible neurocognitive changes would validate their experience, allow better preparation, adoption of self-care behaviour, and ultimately help them cope more efficiently. Participants also felt being informed prior to treatment of such potential late effects prior would mean family/carers could identify, understand, discuss, and better support them if changes in neurocognitive abilities were to develop.

**Required support:***“just a waste of my time and their time, that’s reality*”

The desire to seek support for late effects (supplementary information 3) was in some cases hindered by expectations participants felt they needed to meet. Some felt pressured to have moved on from their cancer diagnosis and be fully recovered from their treatment. Participants felt fatigue or neurocognitive changes were not priorities at follow-up visits with health professionals, with the focus being mostly on potential cancer recurrence and better known physical late effects such as dry mouth or swallowing problems. Participants expressed their impression of healthcare professionals having a poor understanding of, or interest in, fatigue and neurocognitive changes (Table 2, section F, comment 3). This made them feel doubtful healthcare professionals could help with these issues. In this respect, most participants did not feel the support offered during follow-up was adequate (Table 2, F4). Difficulties getting appointments (the further out from the treatment they were), and the quality of consultations were criticised.

Some participants did find support groups valuable, allowing honest conversations and helping to validate their experiences and emotions, thus providing a feeling of reassurance.

## Discussion

The experience of late effects following treatment for OPC is diverse and there is limited data on neurocognition and fatigue in an OPC-only cohort. This is the first study to qualitatively investigate OPC survivors’ experiences of CF and self-perceived change in neurocognitive abilities.

Findings suggest OPC survivors experience fatigue which persists into long-term survivorship. Although the precise pathophysiology behind CF following cancer treatment remains unclear, inflammation, depression, impaired sleep, comorbid conditions, or other late effects are implicated as contributory factors [17], [45], [46], [47]. Yet, similar to other cancer groups and in contrast to international guideline recommendations [48], OPC survivors’ fatigue is not routinely assessed or managed in practice.

In this study, participants experienced severe physical fatigue in the immediate post RT period, with this subsiding as acute RT effects settled. Beyond this timeframe, emotional/cognitive fatigue emerged as the more affected domain in participants with CF. In a review which assessed the multiple-symptom concept of fatigue in a cancer cohort, physical fatigue was reported to increase during anti-cancer therapy, decreasing significantly afterwards [43].That said, the longitudinal course of emotional and cognitive fatigue is less certain, with contradictory findings reported [43]. Emotional and cognitive fatigue in participants usually presented with difficulty concentrating. Similar to patients with chronic fatigue syndrome [49], emotional and cognitive fatigue could be an important correlate of cognitive impairment in OPC survivors, corroborating the multifactorial and multidimensional nature of these constructs, with overlapping elements.

Participants self-reported neurocognitive complaints several years post-treatment. Several domains of functioning were affected in our sample: attention/concentration, processing speed and especially memory. Subjective assessment of short-term memory by participants was in keeping with impairment in working memory. Working memory, which has been widely studied, refers to short-term maintenance of information in the absence of sensory input, and plays a vital role in guiding complex cognitive behaviour [50]. A previous study attributed perceived neurocognitive functioning as an important predictor of communicative outcomes in HNC survivors [32]. The current finding expands further on how attention, memory, and problem-solving affect functioning across several aspects of daily life, including limiting the capacity to return to work following non-surgical treatment. A Finnish study of a heterogenous group of cancer survivors (not including HNC), found 19 % reported deterioration in mental work ability [51]. Paid work is known to boost an individual’s financial, social and psychological health [52] and is an important component of QoL [53]. In this study, support from employers played a vital role in being able to return to work successfully and retain employment in the same job role as pre-treatment. Our data also showed neurocognitive impairment threatened social participation, leisure activities, emotional equilibrium and mood.

Compensatory strategies were self-developed by participants and applied to everyday life. These included memory aids, adopting a slower pace for demanding tasks and relearning, which are integrated coping mechanisms. Retraining, which is the repeated practice of tasks, and compensation, which is focusing on learning new strategies and alternative means to improve daily functioning, form an integral part of cognitive rehabilitation programmes [54]. This has been used successfully to improve neurocognitive outcomes in stroke, traumatic brain injury and brain tumour patients [55], [56], [57], [58]. Participants also relied on physical and psychological support from carers. However, they reported being reluctant to seek assistance to avoid burdening caregivers. Current late effect follow-up services were reported as providing inadequate support for neurocognitive problems and fatigue.

Similar to other studies in cancer patients, there is a perceived lack of adequate information exchange, with improved pre-treatment counselling and information dissemination regarding chronic fatigue and the possibility of neurocognitive impairment being required [59], [60], [61]. For many participants, their experiences differed significantly from their expectations. In an online survey of 403 US patients treated with RT the greatest divergence between pre-treatment expectations and toxicity from cancer treatment related to energy levels, particularly following multimodality therapy [59]. Increased awareness of neurocognitive dysfunction and fatigue, could aid the emotional adjustment of survivors living with these issues and help families understand limitations in what survivors can and cannot do as well as before [62].

This study has limitations. First, all participants received CRT and our findings may not reflect the experiences of patients treated with RT alone. However, it is important to recognise qualitative studies do not aim to produce generalisable findings. Second, we asked long-term survivors to reflect on their whole cancer trajectory, hence there may be some recall bias. This investigation is also limited to patient perspectives only.

Despite these limitations, this study has several strengths. We focused on an OPC-only cohort, which is under-researched. Qualitative methodology allowed us to explore in-depth, the experience and impact of fatigue and neurocognitive changes. It highlights the impact of these changes on personal health, relationships, work, sense of security and social belonging.

Results of this study could enhance patient understanding and increase clinician awareness of the potential risks of post-radiotherapy neurocognitive issues and fatigue. Going forward these risks should be integrated into informed treatment decision-making. Neurocognitive and fatigue assessments should ideally be incorporated into routine follow-up protocols to allow timely intervention, if required. Future research could evaluate the impact of coping strategies, long-term support programs and interventions for managing or mitigating these issues, all with the ultimate goal of optimising quality of life in OPCSs.

## Conclusion

This study provides valuable insight into survivors’ experience of fatigue and neurocognitive changes following RT for OPC. Emotional and cognitive fatigue were most affected, along with cognitive complaints across several domains, with a likely strong correlation between these late effects. Increased awareness of neurocognitive impairment and fatigue as survivorship issues is required. Recognition of these long-term effects has the potential to improve support services and QoL.

**Statements and declaration**

The authors declare that they have no known competing relevant financial or non-financial competing interests to report

**Funding**

ZIE is a Clinical Research Fellow supported by a Cancer Research UK Award (Leeds-Manchester Stella Erdheim Clinical PhD Fellowship – Grant Reference Number 95653122/95653121)

LM is an Associate Professor funded by Yorkshire Cancer Research (award number L389LM).

FB is an Associate Professor supported by Yorkshire Cancer Research (L389FB).

The authors would also like to acknowledge Cancer Research UK funding for the Leeds Radiotherapy Research Centre of Excellence (RadNet; C19942/A28832) and Yorkshire Cancer Research funding of the ROC-oN study.

## CRediT authorship contribution statement

**Zsuzsanna Iyizoba-Ebozue:** Conceptualization, Methodology, Formal analysis, writing, review and editing, Visualisation. **Emma Nicklin:** Conceptualization, Methodology, Analysis, Review and editing. **Robin Prestwich:** Conceptualization, Review and editing. **Sarah Brown:** Methodology, Review and editing. **Emma Hall:** Methodology, Review and editing. **John Lilley:** Methodology, Review and editing. **Matthew Lowe:** Methodology, Review and editing. **David J Thomson:** Methodology, Review and editing. **Finbar Slevin:** Methodology, Review and editing. **Louise Murray:** Conceptualization, Methodology, Formal analysis, Review and editing, Supervision. **Florien Boele:** Conceptualization, Methodology, Formal analysis, Review and editing, Supervision.

## Declaration of competing interest

The authors declare that they have no known competing financial interests or personal relationships that could have appeared to influence the work reported in this paper.
