# Supplementary material for: “Why am I still suffering?”: Experience of long-term fatigue and neurocognitive changes in oropharyngeal cancer survivors following (chemo)radiotherapy
Source: Tech Innov Patient Support Radiat Oncol. 2024 Mar 8;30:100241. doi: 10.1016/j.tipsro.2024.100241 (PMC10951087; doi:10.1016/j.tipsro.2024.100241)
Supplement: Supplementary data 1 [file mmc1.docx]

**Section 1: Interview guide**

**Opening**

**Establish rapport**

Introduce yourself and the project.

**Purpose and motivation**

Explain the aims of the interviews.

**Practicalities**

Explain how long the interview should take and explain they can take breaks whenever needed. Adhere to ethical standards: explain the audio-recording, confidentiality, their right to opt-out without affecting care. Ask if any questions.

*Start audio-recording.*

**Consent to participate**

If a physical consent form has been signed reconfirm the participant is happy to continue and gives their consent to participate. If no physical consent form can be taken then run through the patient consent form with them, asking them to state that they consent to participate. Stop recording following consent and start again before commencing with interview.

**Part 1- Background**

*Can you please tell me a little about you in any way you would like to?*

- Your relationship status/family/children?
- Education background
- What are your interests/what do you like to do in your spare time?

*Can you now tell me more about when you were diagnosed with oropharyngeal cancer?*

- When were you diagnosed?
- Can you tell me what symptoms you had leading up to your diagnosis?
- Can you now detail the treatment you received?

**Part 2 – Fatigue**

*This study is interested in fatigue, and when we say fatigue, we mean a lack of energy and motivation —both physical and emotional.* *What can you tell me about your fatigue levels since diagnosis and treatment for cancer?*

- When did you first notice the fatigue start/change?
- How severe would you say the fatigue is?
- Does fatigue affect your daily life? - work, social, relationships, etc.
- Do you feel your fatigue levels affect your mental wellbeing?
- How do you manage your fatigue? – anything that has helped/not helped
- Have you ever sought/received help? (medical, therapeutic, professional services)
- Can you remember if changes to fatigue was discussed as a side effect prior to treatment? – Essentially were you aware of fatigue as a potential side effect?
- Is there anything you wish you had known?
- What advice (if any) do you have for other patients/caregivers/healthcare providers

**Part 3 – Cognition**

*We are also interested in cognition. Cognition is a term that covers things like memory, thinking and attention. What can you tell me about your memory/thinking/attention since diagnosis and treatment for cancer?*

- When did you first notice any changes in cognition (what they have described)?
- Have these cognitive changes impacted on your daily life? – work, social, relationships etc.
- Do you feel your cognitive challenges affect your mental wellbeing?
- Is there anything that has helped/not helped (compensatory strategies – How do you manage…?)
- Have you ever sought/received help? (medical, therapeutic, professional services)
- Can you remember if changes to cognition was discussed as a side effect prior to treatment? – Essentially were you aware of cognitive changes as a potential side effect?
- Is there anything you wish you had known?
- What advice (if any) do you have for other patients/caregivers/healthcare providers

**Part 4 – other side effects**

*And can you tell me about any other side effects you have had since your diagnosis and treatment?*

- Have these side effects impacted your daily life? - work, social, relationships etc.
- Do you feel these side effects affect your mental health?
- What would you say is the biggest symptom/side effect of your diagnosis/treatment?
- Have you ever sought/received help for these symptoms/side effects?

**Part 5 – Feedback from the Online Cognition Assessment (Amsterdam Cognition Scan)**

*Now if it’s ok with you, we would like to get your experiences of using the online cognition assessment we asked you to complete?*

- How did you find the tool to use?
- How long did approximately did it take you to complete the assessment?
  - Did you find that amount of time acceptable?
- Did you find the tasks interesting?
- Were there any aspects of it you found difficult?
- Did you find accessing the website/assessment ok?
- Did you require any support in using it?
- Do you think there are any advantages or disadvantages to doing a cognition test online instead of face-to-face?
- Do you have any suggestions on how the assessment could be improved?

**End of the interview**

- Is there anything else that you think we need to know?
- Do you have any questions?
- How have you found this interview?

**Section 2: Summary table of interview codes**

|  | Sub-theme | Code |
| --- | --- | --- |
| Unexpected burden of fatigue | When was fatigue first noticed | While having treatment |
|  |  | Immediately after treatment |
|  | Fatigue persisting months beyond treatment | Months after treatment still felt fatigued |
|  |  | Severity and experience of persistent fatigue |
|  |  | Impact of fatigue on daily life |
|  | Perceived causes of persistent fatigue | Perceptions around possible causes of symptoms- age, menopause, thyroid problems, treatment |
|  |  | Factors influencing fatigue- exacerbated by work |
| Neurocognitive changes | Timing of observed neurocognitive changes | Cognitive changes not noticeable right away but became more apparent in long-term |
|  |  | Neurocognitive changes persisted |
|  | Type of neurocognitive changes/domains affected | Concentration/attention |
|  |  | Processing speeds/word finding |
|  |  | Memory |
|  | How neurocognitive changes affect daily life | Performance at work hindered |
|  |  | Social interaction with family and friend negatively impacted |
|  |  | Leisure activities affected– hobbies not as fun anymore and even simple things feels harder |
|  |  | Mental health affected – anxiety, low mood, frustration, fear experienced |
| Other long-term side effects | Physical side-effects | Tooth loss/Fear of tooth loss |
|  |  | Shoulder/neck pain /dystonia |
|  |  | Demyelination/myelin sheath damage |
|  |  | Neuropathy/Neuralgia |
|  |  | Hoarse/croaky voice |
|  |  | Swallow difficulties/ dysphagia |
|  |  | Burning tongue/sore mouth |
|  |  | Change in taste |
|  |  | Problems with intimacy /sexual relationship |
|  |  | Extreme mucus |
|  |  | Dry mouth |
|  |  | Eating/drinking sensitivities |
|  |  | Hearing problems/tinnitus |
|  |  | Issues with balance and gait |
|  | Psychological side-effects | Anxiety/feeling low |
|  |  | Feelings of social isolation and social withdrawal |
|  |  | Affects overall health and well being |
| Navigating Changes | Coping with fatigue in the long-term/How fatigue managed | Adaptive mechanisms- Changing approach to tasks (intense tasks carried out early, splitting up chores) |
|  |  | Lifestyle changes- diet, eating habits, rests |
|  |  | Changes to work patterns- working from home, stepping down to less than full time |
|  |  | Social support systems - from family and work colleagues |
|  | Coping with neurocognitive changes in the long-term/How neurocognitive changes managed | Adaptive mechanisms- noting taking/list /memory aids, slower pace, breaking down tasks so simpler to process |
|  |  | Change to work – change in job role, pattern, career change, retiring early |
|  |  | Social support systems - from family and work colleagues, |
|  |  | Making a daily effort |
|  | Resilient mental approach | Positive actions/attitude to managing side effects. |
|  |  | Looking at the bigger picture –not letting side effects bother you/change in perspective |
| Insufficient awareness | No real awareness of late effects | Felt unprepared for long term side effects |
|  | Information provision about fatigue prior to radiotherapy | Quality and details in information provided- fatigue was not emphasised and glossed over |
|  |  | Expectations of post treatment side effects different from reality |
|  |  | Improvement in information dissemination needed |
|  | Awareness of neurocognitive deficit as a potential long term side effect of treatment | No or very little pre-warning of potential neurocognitive changes as treatment sequalae prior to treatment |
|  |  | Important for information to be given pretreatment |
|  |  | More awareness needed among patients and health care professionals |
| Required support | Inadequate late effects service/ poor experience with follow up | More emphasis on cancer recurrence and very little interest in anything else. |
|  |  | Uncertainty where /how to get professional support |
|  |  | Had to self-seek information - Researched/read about side effects and how to minimise |
|  |  | Fear of recurrence/still need reassurance from check-ups and hard to get appointments. |
|  |  | Support not helpful. No support for carers |
|  |  | GPs not aware |
|  |  | Expectations from family and friends – “you should be alright” |
|  | Support with coping with late effects | Support group/talking to others who have been through the same experiences |

**Section 3: Late effects and participant experience**

| Side Effect | Illustrative quotes |
| --- | --- |
| Tinnitus | *“Tinnitus, which I had before…but it’s off the scale sometimes at the moment….and of course when you get into bed, whatever, you just hear it all the time, and it is, it has been now for the last 6 months, it’s been getting worse” [Participant 8]* |
| Deafness | *“it took 60% of my hearing away, in terms of the higher tones, so that was to adjust to with getting hearing aids” [Participant 12]* |
| Tight jaw | *“my jaw is still quite tight, you know, I can’t open my mouth anywhere near as wide…” [Participant 6]* |
| Dental issues | *“since the treatment I’ve lost a couple of teeth at the same side as I had the radiotherapy…some of them are coming out because, because of the treatment across that area. …. never appeared till about a year down the line after treatment’s finished, and then all of a sudden, I’ve got these wobbly teeth and I’m thinking, what’s going on here, you know” [Participant 4]* |
| Dry mouth | “*The biggest side-effect is, well the ongoing one you live with is lack of salivary glands, they’ve knocked those out and so you’ve got continuously a dry mouth which makes sometimes difficulty when you wake up in the morning, it’s very, very dry……. becomes so dry that when I come in and we have something to eat I can’t even, I can’t think about swallowing for a while, got to get it going again, you know, it takes time. So that’s the worst side-effect” [Participant 8]* |
| Burning tongue | *“I miss swallowing, yeah, but me mouth, I can be in pain. It’s virtually every day, it is every day, it's not often that I get any relief” [Participant 14]* |
| Extreme mucous | “*you also cough up a heck of a lot of mucus, so I produce mucus for fun, ……I always have a hanky or something to hand …. it seems to be worse when you’re exerting yourself, I don’t know why, but the mucus is real, and I can do a box of tissues in 2 days” [Participant 8]* |
| Eating and drinking sensitivity | *“I can’t drink tap water even now; I have to drink bottled water. Our tap water at home is hard, to me it’s pure bleach……No wine, no alcohol at all” [Participant 5]* |
| Dysphagia | *“sometimes just seen muscles not responding, muscles not as strong as it was, I’ve had a couple of occasions where I’ve been eating and talking and probably eating too fast where I’ve, they’ve just got stuck in my throat, that’s happened twice, that’s been a bit scary” [Participant 15]* |
| Voice changes | *“I’ll tell you one thing that I wanted to mention as well …. me voice used to go a lot, it used to go really croaky and disappear…. it sounds awful. No, the only thing that can affect your daily life is me voice, if it goes….” [Participant 7]* |
| Shoulder/neck dystonia | *“my neck kept on going into spasm and I couldn’t move” [Participant 10]* |
| Myelin damage | *“the first time it happened it was literally like a flush down your back and spine into your legs, it was the weirdest thing, and then, you know, I could recreate it by just leaning forward and bending down but as I say over time that’s just tailed away to complete insignificance…. and I knew what it was” [Participant 1]* |
| Neuropathy | *“I get, me nerve endings sometimes twitch which can be quite sore because they’ve been burnt away me consultant said” [Participant 21]* |
| Balance issues | *“if I don’t stand up and wait after about ten seconds of walking, I realise [I need to] hang on a minute, I’ve got to just hold onto something, just to steady myself…. the balance and everything are still a residual effect of all the treatments” [Participant 12]* |
| Sexual intimacy | *“……. all the things like that make a massive difference to how you feel when you want to kiss. Me and {partner]haven't kissed for nine years other than a peck on the cheek” [Participant 18]* |

Supplementary Table 2. Late effects of HNC (Chemo)Radiotherapy
